# Addition of exogenous cytokines in mixed lymphocyte culture for selecting related donors for bone marrow transplantation

**DOI:** 10.1590/S1516-31802002000600004

**Published:** 2002-11-01

**Authors:** Jeane Eliete Laguila Visentainer, Sofia Rocha Lieber, Lígia Beatriz Lopes Persoli, Afonso Celso Vigorito, Francisco José Penteado Aranha, Cármino Antonio de Souza

**Keywords:** Mixed lymphocyte culture, Bone marrow transplantation, Cytokines, Graft-versus-host disease, Cultura mista de linfócitos, Transplante de medula óssea, Citocinas, Enxerto, Hospedeiro

## Abstract

**CONTEXT:**

Mixed lymphocyte culturing has led to conflicting opinions regarding the selection of donors for bone marrow transplantation. The association between a positive mixed lymphocyte culture and the development of graft-versus-host disease (GVHD) is unclear. The use of exogenous cytokines in mixed lymphocyte cultures could be an alternative for increasing the sensitivity of culture tests.

**OBJECTIVE:**

To increase the sensitivity of mixed lymphocyte cultures between donor and recipient human leukocyte antigen (HLA) identical siblings, using exogenous cytokines, in order to predict post-transplantation GVHD and/or rejection.

**TYPE OF STUDY:**

Prospective study.

**SETTING:**

Bone Marrow Transplantation Unit, Univer- sidade Estadual de Campinas.

**PARTICIPANTS:**

Seventeen patients with hematological malignancies and their respective donors selected for bone marrow transplantation procedures.

**PROCEDURES:**

Standard and modified mixed lymphocyte culturing by cytokine supplementation was carried out using donor and recipient cells typed for HLA.

**MAIN MEASUREMENTS:**

Autologous and allogenic responses in mixed lymphocyte cultures after the addition of IL-4 or IL-2.

**RESULTS:**

In comparison with the standard method, average responses in the modified mixed lymphocyte cultures increased by a factor of 2.0 using IL-4 (p < 0.001) and 6.4 using IL-2 (p < 0.001), for autologous donor culture responses. For donor-versus-re- cipient culture responses, the increase was by a factor of 1.9 using IL-4 (p < 0.001) and 4.1 using IL-2 (p < 0.001). For donor-versus-unrelated culture responses, no significant increase was observed using IL-4, and a mean response inhibition of 20% was observed using IL-2 (p < 0.001). Neither of the cytokines produced a significant difference in the unrelated control versus recipient cell responses.

**CONCLUSION:**

IL-4 supplementation was the best for increasing the mixed lymphocyte culture sensitivity. However, IL-4 also increased autologous responses, albeit less intensively than IL-2. Thus, with this loss of specificity we believe that it is not worth modifying the traditional mixed lymphocyte culture method, even with IL-4 addition.

## RESUMO

**CONTEXTO:**

Ensaios de cultura mista de linfócitos têm levado a opiniões divergentes sobre a seleção de doadores compatíveis em transplante de medula óssea. Uma associação entre cultura mista de linfócitos positiva e o desenvolvimento de GVHD (do inglês: graft-*versus*-host disease) é ainda incerta. O uso de citocinas exógenas em cultura mista de linfócitos poderia ser uma alternativa para aumentar a sensibilidade de testes de cultura.

**OBJETIVO:**

O objetivo de nosso estudo foi aumentar a sensibilidade da cultura mista de linfócitos entre doador e receptor irmãos HLA-idênticos, usando citocinas exógenas, com o objetivo de prever GVHD e/ou reações de re-jeição após transplante de medula óssea.

**TIPO DE ESTUDO:**

Estudo prospectivo.

**LOCAL:**

Unidade de Transplante de Medula Ós-sea, Universidade Estadual de Campinas.

**PARTICIPANTES:**

17 pacientes com doenças hematológicas e seus respectivos doadores sele-cionados para o transplante de medula óssea.

**MÉTODOS:**

ensaios de cultura mista de linfócitos padrão e modificada pela suple-mentação com citocinas foram realizados com células de doadores e receptores, previamente tipados para os antígenos HLA. Respostas em culturas foram medidas pela incorpora-ção total de isótopos em contagem por minuto (cpm) após os ensaios.

**RESULTADOS:**

Quando comparada ao método padrão, a média das respostas na cultura modificada aumentou duas vezes com IL-4 (p < 0,001) e 6,4 vezes com IL-2 (p < 0,001), para respostas autólogas do doador; 1,9 ve-zes com IL-4 (p < 0.001) e 4,1 vezes com IL-2 (p < 0,001), para respostas do doador *versus* receptor; foi observado um aumento não significativo com IL-4 e uma média de 20% de inibição da resposta com IL-2 (p < 0,001), nas respostas do doador *versus* indivíduos não-aparentados. Nenhuma das citocinas mostrou diferença significativa em respostas de controles não-aparentados *versus* receptor.

**CONCLUSÃO:**

Entre os ensaios propostos aqui para aumentar a sensibilidade da cultura, a suplementação do meio com IL-4 foi o melhor, uma vez que células dos pacientes tiveram um maior crescimento que no método padrão, usando células do doa-dor ou de indivíduos não-aparentados como estimuladoras. Contudo, IL-4 tam-bém aumentou as respostas autólogas, em-bora de uma forma menos intensa quando comparada a IL-2. Em conclusão, com esta perda de especificidade nós acreditamos que não vale a pena modificar a cultura mista de linfócitos tradicional, nem mes-mo com a adição de IL-4.

## INTRODUCTION

Mixed lymphocyte culturing has been used as one of the tools for selecting recipients and donors in bone marrow transplantation for many years.^1^ Nowadays, molecular biology typing procedures have improved the recipient-donor human leukocyte antigen (HLA) matched selection. Nevertheless, it has been reported that mixed lymphocyte culture responses could be related to chronic graft-versus-host disease (GVHD) in bone marrow transplantation.^2^

The mixed lymphocyte culture assay is based on the recognition of histocompatibility antigens by T cells and their resulting proliferative responses. The *in vitro* test requires the patient's lymphocytes to provide stimulation and to proliferate, although some hematological diseases and their therapies could interfere in such functions. The low responsiveness of some patients could be related to total or partial incapacity to produce and release the cytokines responsible for cell proliferation. Moreover, donor cells could develop weakness in the responses to minor incompatibilities of recipient cells in standard mixed lymphocyte culture assays. Consequently, such assays might not be able to detect the differences between donor and recipient cells that could be important in the development of GVHD. The use of exogenous cytokines in order to increase the mixed lymphocyte culture sensitivity was the main aim of this study. Some of the properties of these cytokines have been shown to enhance the expression of major and minor histocompatibility antigens in stimulator cells, as well as improving the proliferative responses of responder cells.

Interleukin-4 (IL-4) has been described as a lymphoid cell growth factor, which stimulates the growth and survival of certain B-cells and T-cells and induces class II major histocompatibility complex (MHC) expression in resting B-cells and macrophages.^3,4^ Interferongamma (IFN-γ) is a cytokine that can modulate the major histocompatibility complex expression in many lymphoid cells.^5^ Levels of DR-positive human monocyte cells have been seen to be enhanced as earlier as 24 h after incubation with IFN-γ.^6^ Interleukin-2 (IL-2) is a potent lymphoid cell growth factor exerting its biological activity on T lymphocytes and on other cell populations and it also stimulates the synthesis of other T cell-derived cytokines such as IFN-γ.^7^

In order to increase the mixed lymphocyte culture sensitivity, in this study we evaluated the effect of pretreatment of stimulator cells with IL-4 or IFN-γ and the performance of responder cells after IL-2 or IL-4 addition.

## METHODS

Mixed lymphocyte culture tests were carried out using cells from 17 patients (median age: 35 years; 11 males and 6 females) with hematological malignancies. These patients had been selected for bone marrow transplantation procedures from their identical-HLA sibling donors (median age: 36 years old; 10 males and 7 females) at the Centro de Hematologia e Hemoterapia of the Universidade Estadual de Campinas (Unicamp), in 1999 and 2000.

Serological typing for HLA-A and -B was performed using Lambda Monoclonal HLA Class I Tissue Typing Trays^®^ (One Lambda, Canoga Park, CA, USA) and the low resolution molecular typing of DRB1 and DQB1 alleles of the HLA class II locus was performed using Dynal Sequence Specific Primers (SSP^®^, Dynal Ltd., Bromborough, Wirral, UK).

One-way mixed lymphocyte culture assays were carried out using the standard method^8^ and a modified method. Mixed lymphocyte cultures were modified by pretreatment of stimulator cells with cytokines and by addition of cytokines at the beginning of cultures, as described below.

### Pretreatment of stimulator cells with cytokines

Firstly, we determined the best concentration of cytokines capable of increasing the stimulatory capacity of cells in a mixed lymphocyte culture, using 6 unrelated normal pairs. Mononuclear cells from different individuals were incubated with IL-4 (0.1 to 500 ηg/ml; Gibco Laboratories, Grand Island, USA) or IFN-γ (0.1 to 1000 ηg/ml; Gibco Laboratories, Grand Island, USA) at 37° C, in a 5% CO_2_ humidified atmosphere, for 24 hours. After that, these stimulator cell suspensions were washed three times, irradiated and added to plates with responder cells. Plates were incubated for 6 days in a humidified atmosphere with 5% CO . Cultures were pulselabeled with tritiated thymidine and further incubated for 20 h at 37° C. After harvesting by aspiration onto glass fiber filters, total isotope incorporation was determined via the scintillation count per minute (cpm).

### Addition of cytokines at the beginning of cultures

For evaluating the responder cell performance, several doses of IL-2 or IL-4 were added at the beginning of cultures, using 6 unrelated normal pairs. IL-2 (Gibco Laboratories, Grand Island, USA) dose ranged from 0.00001 to 100 ηg/ml and IL-4 (Gibco Laboratories, Grand Island, USA) dose ranged from 0.1 to 500 ηg/ml. Culturing was then performed as previously described. After that, the best concentration of each cytokine was chosen for use in patient-donor mixed lymphocyte cultures.

Thus, the patient-donor mixed lymphocyte cultures were carried out by the addition of IL-2 (10 ηg/ml) or IL-4 (100 ηg/ml) at the beginning of cultures, and then the cultures were performed as previously described.

Comparisons of responses (cpm) in mixed lymphocyte cultures were performed via the Wilcoxon non-parametric statistical method (WinSTAT program, version 3.1 for Windows 95). We considered all p values < 0.05 to be significant.

## RESULTS

### Standardization of pretreatment of stimulator cells with IL-4 or IFN-γ

**Figure 1:**
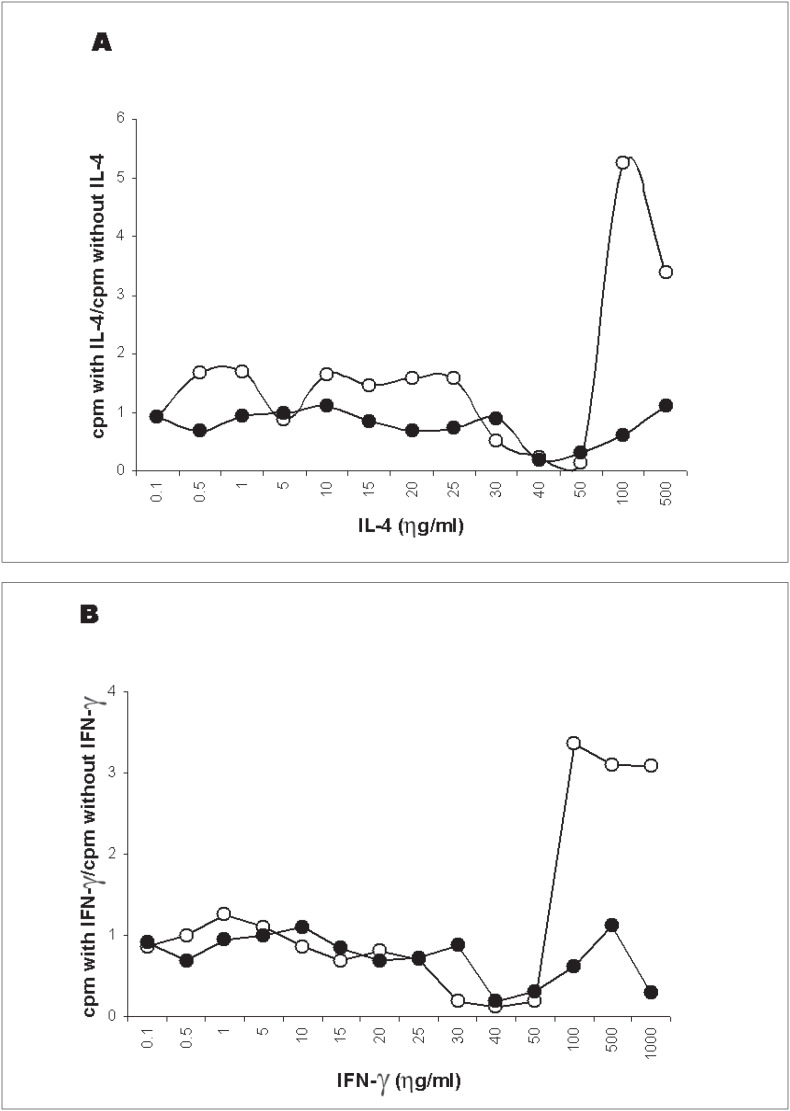
Dose-effect of the preincubation of stimulator cells with IL-4 (A) or IFN-γ (B) on mixed lymphocyte culture (MLC) responses using six unrelated pairs. The results are expressed as the mean cpm observed in the presence of cytokine, in relation to cpm observed in the standard method. Open circles represent autologous MLC and closed circles represent allogenic MLC

The results were analyzed according to the index obtained by the division of the cpm observed in modified mixed lymphocyte cultures by the cpm observed in the standard mixed lymphocyte culture. Figure 1 shows the mean index of mixed lymphocyte culture responses obtained from 6 unrelated normal pairs, using IL-4 (A) or IFN-γ (B) pretreatment of stimulator cells. The pre-incubation of stimulator cells with IL-4 or IFN-γ did not increase allogenic mixed lymphocyte culture responses (closed circles). On the contrary, with rising cytokine concentrations, the autologous responses (open circles) increased significantly. Therefore, these procedures could not be performed in recipient-donor cultures.

### Standardization of IL-4 or IL-2 dose added to the culture

**Figure 2:**
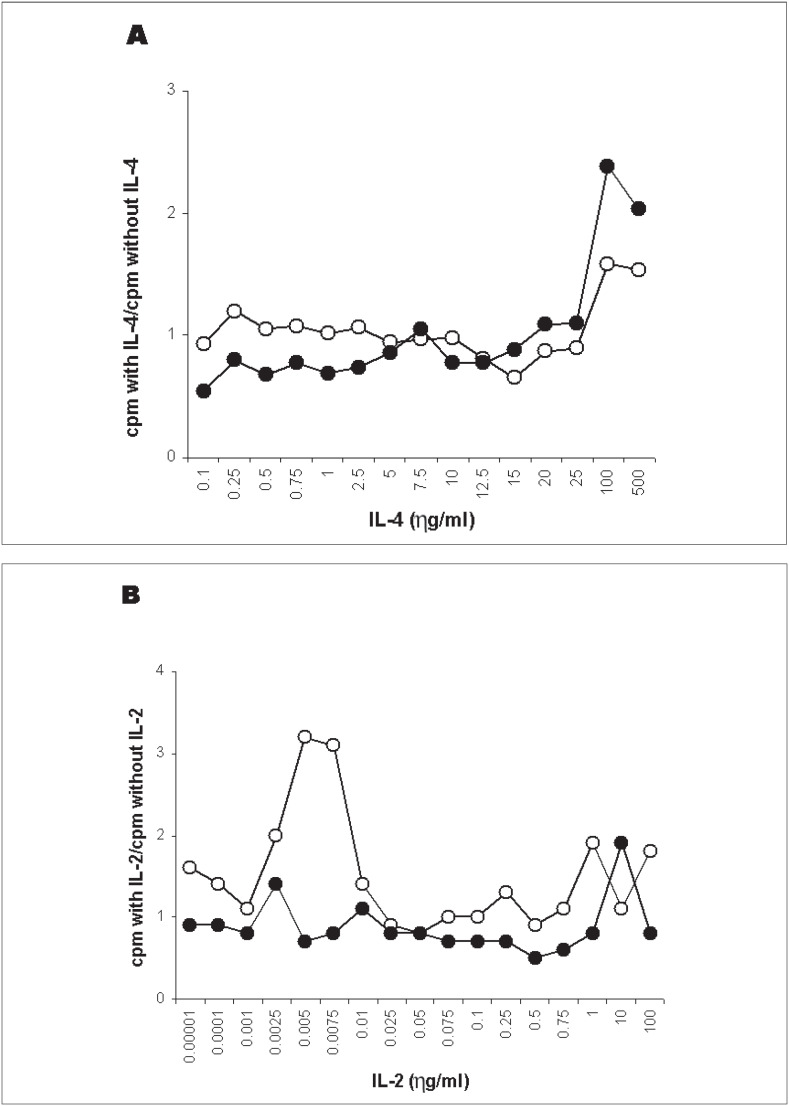
Dose-effect of IL-4 (A) or IL-2 (B) onset addition on mixed lymphocyte culture (MLC) responses using six unrelated pairs. The results are expressed as the mean cpm observed in the presence of cytokine, in relation to cpm observed in the standard method. Open circles represent autologous MLC and closed circles represent allogenic MLC

The analyses and cell pairs used in this procedure were the same described for the pretreatment of stimulator cells with IL-4 or IFN-γ and the results are shown in Figure 2. The best concentration was 100 ηg/ml for IL-4, in which the allogenic response increased by 140% and the autologous response increased by 60% (A). No significant increase was observed by adding IL-2 (B). In fact, this interleukin even increased autologous responses (B). The best dose of IL-2 was 10 ηg/ml, in which there was an increase of 90% in allogenic response and 10% in autologous response. Therefore, it was decided that these concentrations could be used for evaluating the responses between donor and recipient selected for bone marrow transplantation.

### Effect of IL-4 or IL-2 addition on donor versus recipient mixed lymphocyte culture responses

**Figure 3:**
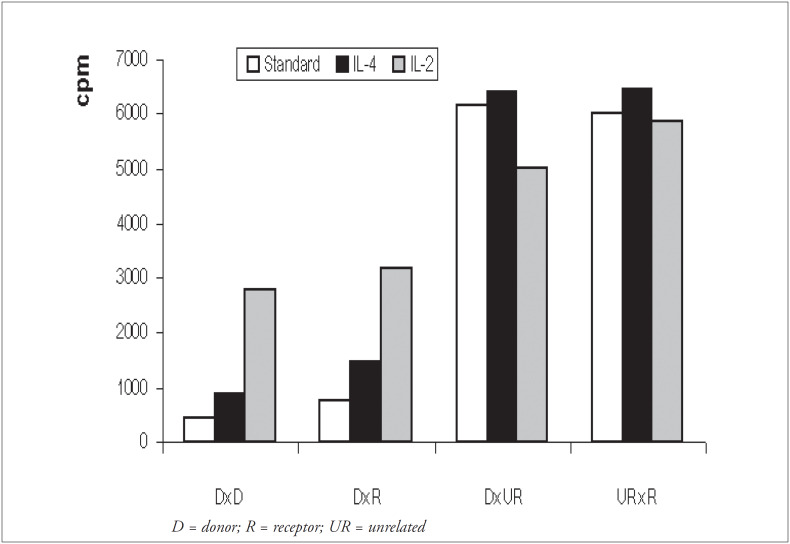
Effect of IL-4 and IL-2 addition on donor versus *donor, donor* versus *receptor, donor* versus *unrelated and unrelated* versus *receptor culture responses. Mean responses in cpm are shown for standard cultures (white column), cultures with IL-4 (black column) and cultures with IL-2 (gray column)*

The mean levels of mixed lymphocyte culture responses (cpm) are shown in Figure 3. In addition to donor-versus-recipient and donor-versus-donor (autologous) cultures, donor-versus-unrelated individual cultures were also included as controls for the response capacity. Unrelated versus recipient cultures were also developed as controls for stimulation capacity from patient cells. In comparison with the standard method, average responses in the modified mixed lymphocyte cultures increased by a factor of 2.0 using IL-4 (p < 0.001) and 6.4 using IL-2 (p < 0.001), for autologous donor culture responses (DxD). For donor-ver- sus-recipient culture responses (DxR), the increase was by a factor of 1.9 using IL-4 (p < 0.001) and 4.1 using IL-2 (p < 0.001). For donor-versus-unrelated culture responses (DxUR), no significant increase was observed using IL-4, and a mean response inhibition of 20% was observed using IL-2 (p < 0.001). Neither of the cytokines produced a significant difference in the unrelated control versus recipient responses (URxR).

### Effect of IL-4 or IL-2 addition on receptor versus donor mixed lymphocyte culture responses

**Figure 4:**
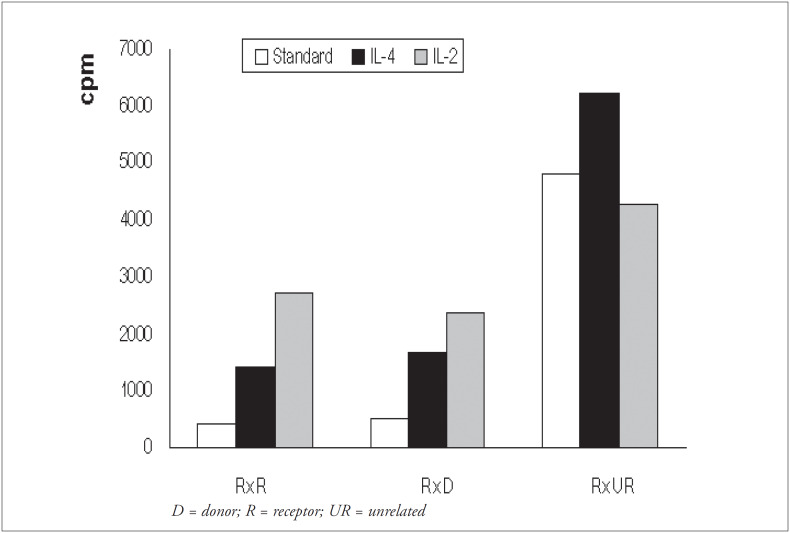
*Effect of IL-4 and IL-2 addition on receptor* versus *receptor, receptor* versus *donor and receptor* versus *unrelated culture responses. Mean responses in cpm are shown for standard cultures (white column), cultures with IL-4 (black column) and cultures with IL-2 (gray column)*

The mean levels of mixed lymphocyte culture responses (cpm) are shown in Figure 4. In addition to recipient-versus-donor cultures, recipient-versus-recipient (autologous) and recipient-versus-unrelated cultures were included, as controls for the response capacity. In comparison with the standard method, the average increases in the modified cultures were by a factor of 3.6 using IL-4 (p < 0.001) and 6.7 using IL-2 (p < 0.001), for autologous recipient responses (RxR). For recipient- versus-donor responses (RxD), the increase was by a factor of 3.1 using IL-4 (p<0.002) and 4.5 using IL-2 (p < 0.001). For recipient-versus-unrelated cultures (RxUR), there was an increase by a factor of 1.3 using IL-4 (p < 0.005), but there was also a mean response inhibition of 10% using IL-2 (p < 0.03).

## DISCUSSION

This study demonstrated that the pretreatment of stimulator cells with IL-4 or IFN-γ was not capable of increasing allogenic responses in mixed lymphocyte cultures. However, our data showed that the addition of IL-2 or IL-4 at the beginning of cultures could increase the allogenic responses, although autologous responses were also stimulated.

Nowadays, the routine use of high-resolution DNA typing techniques for class II MHC allele definition has made mixed lymphocyte culturing redundant for recipient and donor matched selection. Nevertheless, a recent report has shown that mixed lymphocyte culture responses are associated with chronic GVHD in HLA-identical sibling transplants.^2^ One explanation could be the fact that this assay enables the evaluation of the stimulatory capacity of other gene products that are also involved in the development of this disease. Thus, the use of exogenous cytokines could increase the mixed lymphocyte culture sensitivity, considering their capacity for enhancing the expression of MHC and non-MHC antigens and the proliferative responses.

Bishara *et al*. (1994) reported that the pretreatment of stimulator cells with IL-4 and IFN-γ resulted in positive mixed lymphocyte culturing between HLA-identical patient-donor pairs.^9^ In comparison with the traditional mixed lymphocyte culture method, in our preliminary tests, these cytokines were unable to improve the responses. This discrepancy could be partially explained by the fact that, in our experiments, both individuals were normal and unrelated. Thus, it is possible that the MHC antigens were already expressed at a high concentration. Apart from this, the stimulation index is usually higher between unrelated than between related persons and the use of exogenous interleukins may make no difference. Although IFN-γ has been described as a good class I histocompatibility antigen inducer, its role in class II antigens is controversial. Some authors have reported that IFN-γ alone does not enhance the expression of class II histocompatibility antigens in some T cell lines^10^ and that a synergism between IFN-γ and other interleukins may be necessary to obtain this effect.

In spite of autologous responses increasing in the tests, the supplementation of the culture medium using exogenous IL-4 or IL-2 seemed to be more promising. Consequently, for receptor-donor bone marrow transplantation culture pairs, we decided to use the doses of 100 ηg/ml and 10 ηg/ml, respectively for IL-4 and IL-2. From this, a raised allogenic response was found, with minimal interference in the autologous response.

Strikingly, IL-2 increased the autologous responses more than the allogenic responses, and the use of donor or patient cells made no difference. Other authors have also obtained similar results.^9^ The IL-2 receptor expression usually varies according to the cell stage: unprimed or naive cells express different IL-2 receptors as compared with activated cells, and the IL-2 itself interferes in its own receptor switch.^11^ Although this switch is usually antigen-driven, proliferation was observed in the absence of allogenic cells.

Consequently, another stimulus must be present. The probable origin of these antigens is the serum used for supplementing the culture medium. In fact, in our previous laboratory experience using fetal calf serum, the autologous proliferation level was higher. On the other hand, we also noticed that normal unrelated responses were not affected by either IL-4 or IL-2 addition, probably because the stimulus was strong enough to induce maximum response in the standard method, and the serum effect was masked. In fact, in several cases the cpm levels were lower when IL-2 was added to the culture. It is known that chronic T cell stimulation leads to shedding of IL-2 receptors and shed receptor proteins may bind free IL-2, thereby preventing its interaction with cells. The IL-2 is itself involved in such shedding.^12^ Moreover, several cytokines are usually produced *in vitro* and some of them could prevent growth.^13^ The high dose of IL-2 and the long incubation time may induce the release of IL-2 receptors and antagonist cytokines. These events could explain the lower responses, as compared with standard mixed lymphocyte cultures, in which IL-2 quantity is limited to *in vitro* production.

Among the four assays proposed here for increasing the mixed lymphocyte culture sensitivity, the IL-4 supplemented medium was the best. Using IL-4, patients’ cells had greater growth than in the standard method using either donor or unrelated as stimulator cells. IL-4 also increased autologous responses, although in a less intensive way than IL-2 did. Bishara *et al*. (1994) detected an association between positive mixed lymphocyte cultures modified by the addition of IL-4 and acute GVHD.^9^ However, in our study, we could not confirm these results because IL-4 addition was not considered an adequate technique for evaluating responses in mixed lymphocyte cultures.

Although these assays proposed here did not increase the sensitivity of mixed lymphocyte culturing, the development of methods for predicting post-transplantation GVHD and/or rejection has particular importance in allogenic bone marrow transplantation.

## CONCLUSION

Exogenous cytokines did not permit an increase in the sensitivity of mixed lymphocyte culturing, due to loss of specificity. Therefore, we believe that it is not worth modifying the traditional mixed lymphocyte culture method, even with the addition of IL-4.
